# Thermal Tolerance Limits of Diamondback Moth in Ramping and Plunging Assays

**DOI:** 10.1371/journal.pone.0087535

**Published:** 2014-01-27

**Authors:** Chi Nguyen, Md Habibullah Bahar, Greg Baker, Nigel R. Andrew

**Affiliations:** 1 Centre for Behavioural and Physiological Ecology, Zoology, University of New England, Armidale, New South Wales, Australia; 2 Agriculture and Agri-Food Canada, Saskatoon Research Centre, Saskatoon, Saskatchewan, Canada; 3 SARDI Entomology Unit, South Australian Research and Development Institute, Adelaide, South Australia, Australia.; CNRS, France

## Abstract

Thermal sensitivity is a crucial determinant of insect abundance and distribution. The way it is measured can have a critical influence on the conclusions made. Diamondback moth (DBM), *Plutella xylostella* (L.) (Lepidoptera: Plutellidae) is an important insect pest of cruciferous crops around the world and the thermal responses of polyphagous species are critical to understand the influences of a rapidly changing climate on their distribution and abundance. Experiments were carried out to the lethal temperature limits (ULT_0_ and LLT_0_: temperatures where there is no survival) as well as Upper and Lower Lethal Temperature (ULT_25_ and LLT_25_) (temperature where 25% DBM survived) of lab-reared adult DBM population to extreme temperatures attained by either two-way ramping (ramping temperatures from baseline to LT_25_ and ramping back again) or sudden plunging method. In this study the ULT_0_ for DBM was recorded as 42.6°C and LLT_0_ was recorded as −16.5°C. DBM had an ULT_25_ of 41.8°C and LLT_25_ of −15.2°C. The duration of exposure to extreme temperatures had significant impacts on survival of DBM, with extreme temperatures and/or longer durations contributing to higher lethality. Comparing the two-way ramping temperature treatment to that of direct plunging temperature treatment, our study clearly demonstrated that DBM was more tolerant to temperature in the two-way ramping assay than that of the plunging assay for cold temperatures, but at warmer temperatures survival exhibited no differences between ramping and plunging. These results suggest that DBM will not be put under physiological stress from a rapidly changing climate, rather access to host plants in marginal habitats has enabled them to expand their distribution. Two-way temperature ramping enhances survival of DBM at cold temperatures, and this needs to be examined across a range of taxa and life stages to determine if enhanced survival is widespread incorporating a ramping recovery method.

## Introduction

There are strong evolutionary and ecological relationships between living organisms and their natural environment [1]. As poikilothermic organisms, insects are strongly influenced by the surrounding climatic components (e.g. temperature and relative humidity) [2], [3], which have a direct influence on their metabolic rate, activity patterns, and development [4]. Among those components, temperature plays a key role in insect life history [5]. Environmental temperature has varied and complex impacts on insects; therefore, the seasonality and evolutionary responses of insects can be influenced by temperature over a range of time scales from minutes to centuries [6]. Further, climate change will influence geographical ranges, population dynamics, and the life cycle of many organisms [7]–[9]. Consequently, faced with unfavorable thermal environments, insects may maintain their key activities or improve survival in their current range using physiological adjustments [5], [10].

Physiological adjustments to body temperatures can be used independently, or in conjunction with behavioural adjustments to deal with extreme temperature exposure [11]. Alteration of tolerance to extreme temperatures can occur at a range of timescales. At short time scales (exposure for a few hours) codling moths exposed to 43°C for 2 hours increased survival from 20% to 90% [5]. At diurnal timescales, the supercooling point of three Antarctic Collembola species exhibited a range of supercooling point distributions between day and night [12]. As well, diurnal temperature variation over two days enhanced heat shock survival and reduced cold shock survival of *Drosophila melanogaster* [13]. At longer, seasonal time scales, a field fresh population of larval codling moth in Central Europe reduces its supercooling point from −15.3°C in summer to −26.3°C in winter [14] and in Iran from −12.4°C in summer to −20.2°C in winter [15].

For insects without pre-exposure to treatment temperatures, survival changes between more extreme temperatures and longer durations. For example the laboratory reared adult codling moth, *Cydia pomonella* exhibited at range of lethal temperatures (between −20°C and −5°C for lower temperatures and 32°C and 47°C for upper temperatures) when exposed to these temperatures for times ranging between 30 minutes and 4 hours [5]. The meat ant *Iridomyrmex purpureus* collected from Armidale, Australia also showed substantial variation in survival rates when exposed to temperatures between −18°C and −11°C, and 43°C and 50°C for times ranging between 5 minutes and 4 hours [16]. Survivability also differed between seasons, and between laboratory acclimated and field fresh specimens [16].

Insect responses to extreme temperatures also has an influence on species abundance and distribution. The ability to survive extreme low-temperature environments via tolerating or avoiding freezing using a range of strategies is a key to overwintering at higher latitudes [6], [17], and can play a key role in enabling species to increase (or decrease) their distributions under human-induced climate change conditions. However, if insects are exposed to cool weather precipitation at overwintering sites [18] or if overwintering capabilities are lost when insulating snow cover is replaced by exposure to frost [19], populations can be driven locally extinct, or restricted from areas via key life-history stressors. Species may also have a range of physiological limits, dependent on their distribution. For example, *Drosophila i*n the tropics have a limited response to evolve increase desiccation and cold resistance, temperate species are more cold resistant, desert species are desiccation resistant and cosmopolitan species have a high level of resistance to all stressors [20]. Due to such a range of survival rates between time and temperature exposure interactions, as well as hardening, acclimation, and nutritional influences on thermal tolerances, it is difficult to obtain a ‘universal’ thermal limit [21] that can characterise a species survival response within its local environment that can be extrapolated across its entire distribution. These thermal limit influences are further exacerbated when pest species populations, or native species that move into novel environments with a changing climate (either by translocation or by natural migration), go through a ‘niche shift’ by adapting to these novel environments [22].

Knowledge about the relationship between temperature and physiological limits of insects is necessary to predict their adaptability and population growth in terms of climate change. This is particularly true when assessing responses of insect pests. Nevertheless, almost all studies dealing with the effects of temperature on insect pests have been conducted at constant temperatures [23]–[25], which are not comparable to natural field conditions and it becomes difficult to define a universal thermal limit [21].

Recent studies indicate that the experimental protocol (i.e. constant extreme temperatures vs. ramping temperature assays) influences the thermal limits of insects [26]–[29]. Incremental changes in temperature, or ‘ramping assays’ are considered ecologically relevant as they allow time for expression of physiological coping mechanisms [21]. However the amount of time that ectotherms are kept in thermal tolerance assays may reduce physical fitness, which could negate any beneficial responses, such as thermal acclimation [28]. In addition, issues relating to starvation and desiccation can also arise when insects are exposed to ramping methods, however these impacts may be unpredictable between ramping methods and taxa studied [29]. According to Jensen's inequality, environmental variance can exert important effects on patterns and processes in nature that are independent of average conditions [30]. For example, fluctuating compared to constant temperatures caused shorter development times in the butterfly *Lycaena tityrus* (Lepidoptera: Lycaenidae) [25]. In addition the development time of the diamondback moth (DBM), *Plutella xylostella* was significantly shorter at a fluctuating 7°C (0 – 14°C) than at a constant 7°C [27]. Natural conditions are characterized by daily thermal cycles; gradual temperature changes might affect the biology and interaction between arthropod herbivores, their natural enemies and host plants [22]. Therefore, natural ramping temperatures may provide a more accurate assessment of insect developmental biology than constant temperatures. The standard method of ramping temperatures has been to ramp the temperatures (generally between 0.1°C and 1°C/min) to the end point temperature, and then remove the animals immediately from the end-point temperature [31]–[33], essentially giving the animal a temperature ‘shock’ at their most vulnerable stage of heat exposure. In this study we ramped the temperature back to the starting temp (termed *2-way ramping*) to reduce any potential effects of rapid temperature change when they are in a highly stressed state. A similar method was used by Sinclair *et al.* [34] on the sub-Antartcic caterpillar *Pringleophaga marioni* where caterpillars were placed in a waterbath at 1.6°C, cooled at 0.1°C/min, held for 15 hours (and 5 hours at −18°C) and then rewarmed at 0.1°C/min to 1.6°C to assess recovery from freezing and metabolic rate. Current global climate change models predict an increase in the frequency and severity of extreme temperature events [35], and a temperature ramping rate of 0.25°C/min to this state and then reducing at the same rate does enable an assessment of realistic responses to thermal stress. The ability of an organism to survive under extreme conditions is a significant component of fitness [36].

The diamondback moth (DBM), *Plutella xylostella* (L.) (Lepidoptera: Plutellidae), is one of the most destructive insect pests of cultivated crops worldwide, and primarily feeds on host plants in the Brassicaceae [37]–[39]. DBM is multi-voltine: the species can complete three to four generations per year in temperate regions, and as many as 20 per year in tropical regions [40]. A range of studies have provided valuable information about the relationship between development and temperature of DBM [41]–[44]. However, these studies have not assessed the relationship between the survival rate of DBM and temperature. Although the adult stages are critical for reproduction and dispersal, the thermal tolerance limits of DBM adults is unknown.

The response of larval and pupal DBM to various temperature stresses has been tested in various forms previously. The development of DBM at constant temperatures, assessed by Golizadeh *et al.* [41] ranged from 10°C to 35°C. They found the low temperature threshold of DBM to be 7.06°C when reared on *Brassica oleracea* va *botrytis*. They found no newly hatched larvae would survive to second instar at 35°C. DBM developed successfully using alternating temperatures ranging from 4 to 38°C, as studied by Liu *et al.* [42], but with constant temperatures developed successfully at a lesser range from 8 to 32°C. There were no differences in the temperature tolerance of temperate and tropical DBM populations, at 35°C hatching success was lower than between 15°C and 32.5°C, and at 32.5°C larval survival was lower than between 15°C and 30°C [43]. In 1983, Yamada and Kawasaki [17] assessed the population dynamics of DBM, including hatching and pupation events at benign temperatures with low hatching and pupation rates at the extreme temperatures tested (17° and 30°C). This suggests that adults can survive higher temperatures than larvae, but critical biological processes at all stages, such as egg production, pupation and dispersal is compromised when animals are under extreme stress [7], [8].

To predict the future activities of DBM in a changing global climate, studies of its thermal physiology/tolerance mechanisms are required [7]. In the first instance an assessment of an insect's ability to tolerate extreme temperatures when exposed to them at different rates is required to provide more ecologically meaningful estimates of thermal tolerance [7]. The aim of this study was to determine the ability of DBM to survive in low and high extreme temperatures attained via 2-way ramping and plunging experiments. The study also assessed the influence of the duration of exposure to upper and lower temperature extremes on the survival of DBM.

## Materials and Methods

### Insects

For this study, initial DBM adults were obtained from a stock culture reared at the Waite Campus of the University of Adelaide for approximately 15 years at approximately 24°C without exposure to insecticides (SARDI, Adelaide, SA, Australia). Even though they are effectively a lab colony, and will have lost much of their genetic diversity, we believe there is substantive biologically relevant information that can be inferred from using such a population, to enable comparisons with field fresh DBM populations and with other widely distributed species. Field DBM populations from 46 latitudes have exhibited constant temperature responses [43], and field resistance in DBM has occurred to all available insecticides [37]. This homogeneity could be due to high gene flow between the populations that disrupts local adaptation. The colony of the DBM, was maintained under controlled conditions at 25°C (±2°C), 60±10% relative humidity, and L:D 14∶10 h photoperiod on cabbage plant (*Brassica oleracea* var. *capitata*) for the pre- experimental treatment period at the University of New England, Armidale, NSW, Australia.

### LT_25_ (Lethal Temperature, 25%)

To determine the Upper and Lower Lethal Temperatures (ULT_25_ and LLT_25_ respectively) [45], [46] at each set temperature, 25 individuals per treatment were placed into groups of five DBM adults of mixed gender (individuals were chosen randomly to generalise results for adults, rather than for a specific sex) into each of five clear plastic cylindrical vials (8 cm tall ×2.5 cm diameter) with a waterproof cap. Also adult age, gender and feeding status were not strictly controlled for, to address these potentially confounding factors, but rather to randomise them across the treatments [46]. The vials were held in a frame and plunged into a Grant water bath (GP200-R4, Grant instruments, Cambridge, UK; accuracy ±0.01°C) for the requisite temperature for two hours. In LLT_25_ experiments, a mixture of glycol and water (1∶1 ratio) was used to enable water baths to operate at sub-zero temperatures without freezing. The water baths were set to five temperatures: −13°C, −14°C, −15°C, −16°C and −17°C. For ULT_25_, the water baths, using distilled water, were set to four temperatures: 40°C, 41°C, 42°C and 43°C. Temperature ranges assessed were based on pre-treatment trials of 100% and 0% survival. Throughout the experiments a gauge copper-constantan thermocouple was placed inside one of the vials and connected to a Squirrel data logger (Squirrel 2020; Grant Instruments; accuracy: ±0.025%) to continuously measure the vial ambient temperature and thereby ensure it was reaching the water bath temperature. After 2 hours of treatment, the vials were removed from the water bath and placed in a constant temperature chamber (25±2°C) for 24 hours and the insect survivorship then recorded. If any individual moth was found to move normally, it was recorded as a survivor [32].

The LT_25_ is defined as the temperatures where 25% individuals survived after 2 hours of exposure to low or high temperatures. The proportion of moths surviving at each temperature was then used to generate a probit model for the proportion of animals alive after a 2-h exposure to a range of low temperatures in SPSS PASW Statistic, version 18 [47] and then curves of upper and lower discriminating temperature were generated using SigmaPlot 12.0 [48]. This is a standard measure for assessing upper and lower lethal temperatures [16], [32], [46].

### Ramping treatments with various durations

#### Cold Shock Survival

**Figure 1:**
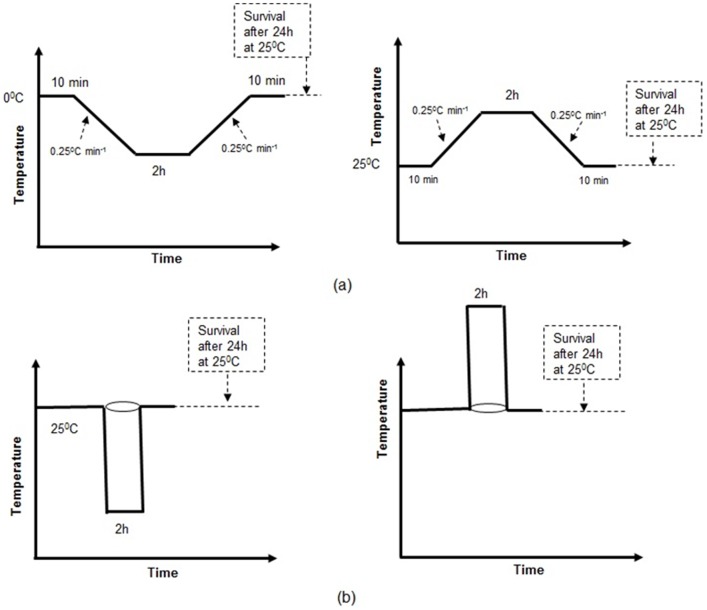
Schematic diagram of experimental protocols used for: (a) two-way ramping temperature assay, (b) plunging temperature assay.

For each ramping treatment, an individual adult moth was placed into one of five clear plastic cylindrical vials (8 cm tall ×2.5 cm diameter) with a waterproof cap. The five vials were held in a frame and plunged into a Grant water bath (GP200-R4, Grant instruments, Cambridge, UK; accuracy ±0.01°C) containing 1∶1 water: glycol mix. The water bath was programmed to decrease the temperature at 0.25°C min^−1^ starting from 0°C. The ramping rate used is known to affect insect thermal tolerance, and we chose to use a ramping rate of 0.25°C min^−1^ as it has been used previously for a range of taxa [10], [45], [49]. After placing the vials containing the moths into the water bath, the moths were given 10 minutes to equilibrate at 0°C before temperature ramping started. The four cold end-set temperatures for ramping were −5°C, −10°C, −15°C and −20°C. Upon reaching the cold end-set temperature, each temperature treatment was kept for one of four holding- time periods (10, 30, 60 or 120 minutes) before the temperature was ramped up to 0°C. Again, the moths were kept 10 minutes to acclimatize to 0°C after temperature ramping up (Fig 1a). The moths were then removed from the water bath and placed in a controlled temperature chamber (25±2°C) for 24 hours, and their survivorship was recorded. Each of the 16 treatments (four cold end-set temperatures x four holding-times) used five adult moths per treatment. A χ^2^ test was carried out to test for significant differences in the number of individual DBM surviving between all 16 temperature/exposure time treatments using a 4×4 matrix (four temperature treatments and four times exposure times; d.f. = 9). The observed surviving DBM in the temperature exposure time treatments were compared to the expected survival number for each treatment (all five individuals).

#### Heat Shock Survival

These experiments were carried out in the same way as the cold shock survival treatments, but using higher temperatures and the GP200-R4 water bath contained 100% distilled water. Heating increments (0.25°C min^−1^) were programmed, starting from 25°C. Once vials were plunged into the water bath, insects were given 10 minutes to acclimatize to 25°C before temperature ramping started. The four hot end-set temperatures were 40°C, 42.5°C, 45°C and 50°C. Once the target temperature was reached, the water bath temperature was maintained at one of four holding temperatures (10, 30, 60 and 120 minutes) before temperature ramping down to 25°C, and then kept for 10 minutes to normalize at 25°C (Figure 1a). Upon completion of the treatments, the vials were removed from the water bath and placed in a temperature controlled chamber (25±2°C) for 24 hours and their survivorship was recorded. Likewise, as for the cold shock survival, there were five adult moths per treatment. A χ^2^ test was carried out to test between all 16 temperature/exposure time treatments using a 4×4 matrix (four temperature treatments and four times exposure times; d.f. = 9). The observed surviving DBM in the temperature exposure time treatments were compared to the expected survival number for each treatment (all five individuals).

### Two-way ramping versus plunging temperature

This experiment compared the DBMs tolerance to cold and heat by measuring their upper and lower lethal temperatures (ULT_25_ and LLT_25_ respectively) when exposed to equivalent minimum and maximum temperatures by ramping (gradual) versus plunging (instant) methods. Five DBM adults were placed into each of five clear plastic cylindrical vials (25 individuals/treatment). The moths were exposed to the range of stressing temperatures using previously described methods. For the ramping treatment a constant rate of cooling or heating was used (0.25°C min^−1^), starting from 0°C for LLT_25_ and 25°C for ULT_25_. After placing the vials containing the moths in the water bath, moths were given 10 minutes to acclimatize to 0°C for LLT_25_ and 25°C for ULT_25_ before temperature ramping started. The minimum temperatures for LLT_25_ were set as −13°, −14°, −15°, −16°, −17°, −18°, −19° and −20°C, and the maximum temperatures for ULT_25_ were set as 40°, 41°, 42° and 43°C. After reaching the target temperature, moths were kept at this temperature for two hours and then ramped back. Again, the moths were kept 10 minutes to normalize to 0°C for LLT_25_ and 25°C for ULT_25_ after temperature ramping back. The vials were then removed from the water bath and the moths held in a climate chamber (25±2°C) for 24 hours and the survivorship was recorded.

For the plunge treatment, the vials were plunged directly into the stress temperatures in the water bath for 2 hours. The stress temperatures ranged from 40°C to 43°C for ULT_25_ and from −13°C to −20°C for LLT_25_ (Figure 1b).The proportion of moths surviving 24 hours after treatment from the ramping experiments, and from the comparison of the ramping and plunging methods were analyzed using a generalized linear model (GLZ) with a binomial distribution and a logit-link function in R [50], [51].

## Results

### Lethal limits - ULT_25_ and LLT_25_

All moths survived at the 40°C treatment, and based on survivorship curves, their survival declined sharply with 80% survivorship at 41°C, and only 25% survivorship at 41.8°C (ULT_25_). Survivorship curves estimate all moths died at 42.6°C (ULT_0_). Conversely, all moths survived a two hour exposure at −12.8°C and there was no survival at −16.5°C (LLT_0_). Based on the survivorship curves, the lowest temperature at which 25% moths survived was −15.2°C (LLT_25_).

### Ramping treatments with various durations

#### Cold Shock Survival

**Figure 2:**
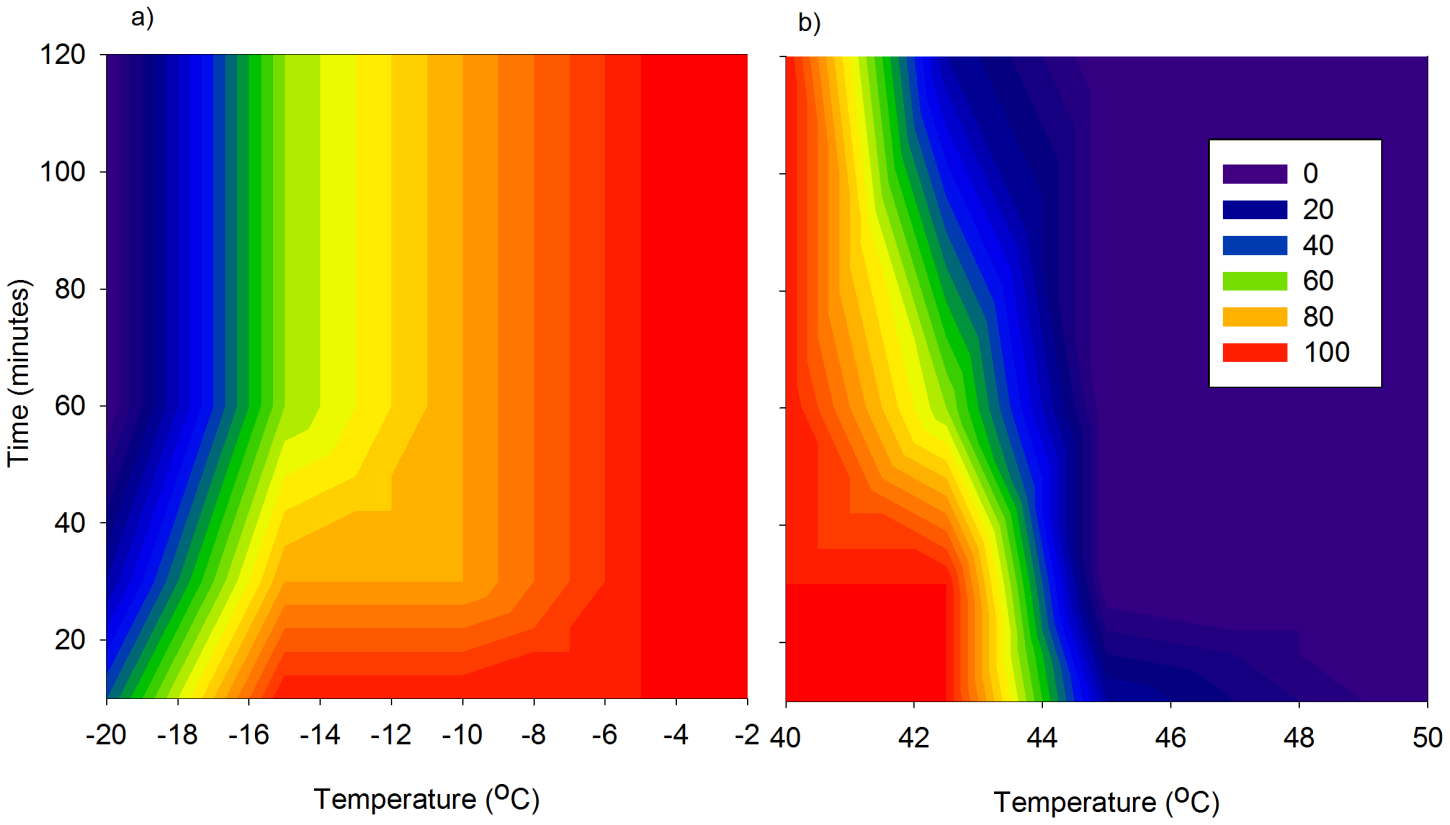
Contour plot of the relationship between the survivorship (%) of DBM (*P. xylostella*) at (a) low temperatures with duration of exposure (10 minutes, 30 minutes, 60 minutes and 120 minutes) and (b) high temperatures with duration of exposure. Survival is indicated by different colour shades (blue  = 0% survival, red  = 100% survival).

Temperature and exposure duration had a significant effect (χ^2^ = 17.4, d.f. = 9, *P* = 0.0423) on the survival of DBM in cooler temperatures. As expected, temperatures below −10.0°C caused increased mortality of DBM (Figure 2a) during all exposure times. The lowest survival occurred at −20°C. When DBM were exposed for 10 minutes at different low temperatures, there was 100% survival until −15°C, with 40% survival at −20°C; survival substantially decreased when exposed to low temperatures for >1 hour (Figure 2a).

#### Heat Shock Survival

At high temperatures there was significant reduction in survival of DBM as temperatures and the duration of exposure increased (χ^2^ = 42.1, d.f. = 9, *P*<0.0001). When DBM were exposed for 10 or 30 minutes at 42.5°C, 100% moths survived, whilst at 60 minutes survival was 60%, and at 120 minutes survival was 20%. The percentage of DBM survivorship was reduced to 20% after only 10 minutes exposure at 45°C, and all moths died when held for ≥30 minutes at this temperature (Figure 2b).

### Two-way ramping versus plunging treatments

**Figure 3:**
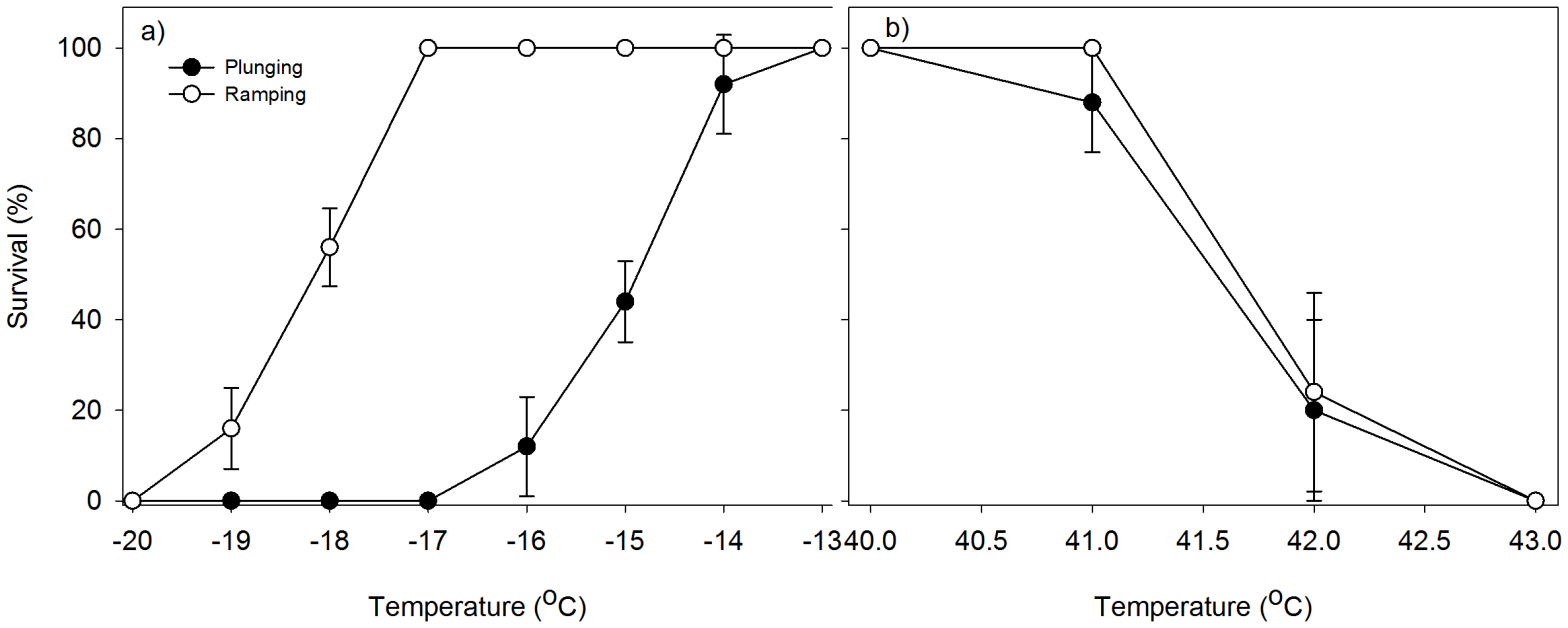
Mean survival (%) of DBM (*P. xylostella*) exposed to plunging and two-way ramping (a) cold temperatures and (b) hot temperatures.

Extreme temperature exposure significantly reduced DBM survival in both plunging and two-way ramping treatments (Figure 3; Table 1). Survival of the DBM adults differed significantly (*P*<0.001) between the plunging and two-way ramping methods. In the two-way ramping treatment, DBM survived at a larger range of temperatures compared to the plunging assay. All the DBM adults survived at temperatures between −13°C to −17°C in the two-way ramping treatment, whereas in the plunge treatment survival declined below −13°C. No survival of DBM occurred when directly plunged at −17°C, and when the temperature two-way ramping treatment reached −20°C (Figure 3a). When exposed to 40°C, 100% of the DBM adults survived both treatments (Figure 3b). At 41°C, the DBM adults survived at the two-way ramping temperature assays but their survival declined substantially when exposed to the direct plunge treatment. At 42°C survival reduced drastically in both treatments, and all the adults were dead at 43°C in both treatments (Figure 3b). Overall there was no significant difference between the effects of plunging and two-way ramping temperatures in the high temperature assay (Table 1b – Plunge vs. 2-way ramping).

**Table 1:** Statistical output for DBM survival when exposed to (a) Low and (b) High temperatures via plunging and 2-way ramping methods. Significant values in bold.

(a) Low Temperatures	Df	Wald χ^2^	P
Plunge vs 2-way ramping	1	78.5	**<0.0001**
Temperature	1	422.86	**<0.0001**
Interaction	1	0.04	0.8498

(b) High Temperatures	Df	Wald χ^2^	P
Plunge vs 2-way ramping	1	0.146	0.702
Temperature	1	270.364	**<0.0001**
Interaction	1	0.093	0.7603

## Discussion

### Lethal thermal limits in adult DBM

Lethal thermal limits (LT_25_) are fundamental physiological traits, and their measurement is receiving increasing attention for identifying species distribution boundaries and responses to climate change [7]–[9], [52]. The ULT_25_ for short-term exposures is between 40 and 50°C for many insects; however, this lethal temperature range may be different among species from particular habitats [53]. Lower lethal temperatures are much more varied [54]. While there is no previously published data on adult DBM surviving below −5°C, Gu [55] showed an Australian DBM population could survive at −5°C for 20 days. At high temperatures, a DBM adult population from eastern China partially survived at temperatures up to and including 40°C [42]. Also in China, DBM adults have been shown to survive single thermal shock events (temperatures at 40°C for up to five hours), but reduced female egg production and subsequent egg hatching [56]. In this study, the LLT_25_ and ULT_25_ were −15.2°C and 41.8°C respectively. As insects are poikilothermic, their body temperature varies with the surrounding temperature, and will strongly influence their motor activity, growth and other physiological processes [2], [57]. In addition, insects are sensitive to changing environmental cues, and many can modify their behavior in response to such changes [58]. The adult life-stage of insects is responsible for dispersal and reproduction but, most work examining thermal tolerance of DBM has not explicitly focused on the adult stage. Therefore future DBM abundance and distribution in Australia will not be substantively reduced with a warming climate as their distribution was expanded markedly over the last few decades due to the greater production of *Brassica* vegetable and oilseed crops across most zones of the continent [37].

### Temperature exposure and duration

We assessed responses to extreme temperature exposure and duration of holometabolous DBM adults. Unlike many insects, DBM does not diapause at any life stage; rather the adult moths may migrate to avoid unfavorable temperatures, particularly low temperatures [59]. The minimum temperatures in winter in most of the agriculture areas in Australia (few areas in Australia have 10% of minimum temperatures any lower than −3°C and these only occur for a short period at night) [60] would not be lethal to DBM. A limitation of DBM abundance is availability of host crops [55]. Our findings of adult moth survival across a wide range of short term temperature fluctuations will assist in predicting DBM outbreaks given the available host crops of DBM in winter and meteorological data. Similar studies are needed on field collected adults as well as other life stages to reveal how those stages are affected by temperatures and in actual field conditions.

There was a rapid decline in DBM survivorship at higher temperatures in that >80% of the moths survived 2 hours of exposure to 41°C, but, based on the survivorship curves, all died when exposed to 42.6°C. Insect death at high temperatures may result from various factors. For example, proteins may denature and clump together at high temperature, and cellular function is affected by changing membrane configuration, intracellular physicochemical condition and protein conformation [53], [61], [62]. In contrast, exposure of insects to low temperature as well as cold shock is reported to cause cellular injuries at freezing point [63], metabolic collapse [64], the loss of extracellular ion-homeostasis [65] and nerve injuries [66].

In the present study, the relationship between temperature and duration of exposure was more complex at higher temperatures than at lower temperatures. That is, the duration of exposure at higher temperatures had a highly significant impact (P>0.0001) on survival, but only marginally significant (P = 0.0423) at lower temperatures. We speculate that at higher temperatures the severity of tissue injury which could not be repaired was probably higher [5], and cell damage would occur [67]. In contrast, Chapman [53] found that the duration of exposure affected insect survival at low temperatures.

### Significance of ramping and plunging protocols

An important outcome of this study was the differences in thermal limits under the plunging direct and 2-way ramping treatments at low temperature, but no differences at high temperatures. In the ramping experiment, the temperatures of the chambers were decreased at a steady rate of 0.25°C min^−1^, held for two hours, and then increased at the same rate whereas in plunge treatment insects were exposed directly to the target extreme temperatures for two hours. Mortality of DBM adults increased more quickly in direct plunge treatment than in the slowly ramping treatment at low temperatures. There was also wider range of temperatures at which DBM adults survived in the 2-way ramping treatment, but not for higher temperatures. For instance, DBM survived 100% at −17°C with ramping method while all moths died when direct plunging in the same temperature. This is not unexpected and these results support previous studies with different insects [5], [26], [45], [61], [68]. These studies suggest that slow rates of temperature change enable a form of rapid cold hardening, which results in an increase survival of arthropods. Slowly ramping the temperature changes may be more ecologically relevant compared to a direct plunge, but ramping enables hardening [62] and may enable other factors to influence survivorship: such as nutritional status and adult age. Therefore, assay techniques should be considered before recommending the laboratory findings of insect threshold temperature in real field conditions [21].

Rates of temperature change and starting temperatures are known to have impacts on critical thermal limits [45], with Terblanche and colleagues finding the Tsetse fly performed worse when temperature ramping occurred at a slower rate for both high and low temperatures. In our experiments, with two-way ramping temperatures both to the end point, and then back to the initial start temperature, for both upper and lower temperatures, we found DBM responded similarly to ramping and plunging at high temperatures, and substantially better to ramping at low temperatures (Figure 3). We expected our ramping treatment to be much more detrimental to DBM survival. In previous temperature ramping tests, the temperature is usually only ramped until the temperature reaches the critical thermal limit, then insects are removed from the experiment and allowed to recover; e.g. [16]. We added an extra ramping recovery protocol back to the start temperature, compounding the temperature stress. We expected this extra stress to increase mortality, but against predictions, DBM showed enhanced survival at the cooler temperatures, and similar survival at upper temperatures, compared to the plunging treatment. This extended exposure time via two-way ramping may give the DBM adults mechanisms to physiologically respond when returning body temperatures to more benign conditions, compared to immediate exposure to benign conditions, particularly at low temperatures.

### Conclusion

As adult thermal sensitivity (upper and lower) found in this study is much broader than ambient air temperatures that they currently have, or are forecasted, to be exposed to under predicted climate change scenarios, we suggest that DBM adults will not be put under extreme physiological stress from a rapidly changing climate. Different thermal tolerance limits of DBM using the two different bioassays, especially at lower temperatures suggests that two-way ramping (ramping to low temperatures and then ramping back to the start temperature) has a positive influence on survival, even if this does increase total exposure time to extreme cold temperatures. Post-exposure recovery to extreme conditions using temperature ramping to recovery temperatures needs to be tested on a range of other taxa to identify if survival is enhanced, particularly when exposed to low temperatures.
